# Mechanisms of systolic heart failure in PP2CxPP5 double transgenic mice

**DOI:** 10.1007/s00210-026-05671-5

**Published:** 2026-07-08

**Authors:** Rebecca Schwarz, Katarina Hadova, Jan Klimas, Igor Buchwalow, Joachim Neumann, Ulrich Gergs

**Affiliations:** 1 Institute for Pharmacology and Toxicology, Medical Faculty, Martin Luther University Halle-Wittenberg, Halle (Saale), Germany; 2 Department of Pharmacology and Toxicology, Faculty of Pharmacy, Comenius University Bratislava, Odbojárov 10, 832 32 Bratislava, Slovak Republic; 3 Institute of Hematopathology, Fangdieckstr. 75a, 22547 Hamburg, Germany; 4 Educational Resource Center for Molecular Morphology, Peoples Friendship University of Russia, Miklukho-Maklaya Str.6, RU-117198 -Moscow, Russia

**Keywords:** Transgenic mice, PP2C, PP5, Heart failure, Hypertrophy, Fibrosis, Inflammation

## Abstract

The function importance of serine threonine phosphatases (PP) in the heart is still subject of investigations. For instance, PP5 and PP2C occur in the human heart. It is unclear how they interact in the human heart. In order to obtain a model system, we have crossbred mice with heart-specific overexpression of PP2C or PP5 to obtain wild-type mice (WT), PP2C transgenic mice (PP2C-TG), PP5 transgenic mice (PP5-TG), and PP2CxPP5 double transgenic mice (DT). Moreover, while PP2C-TG and PP5-TG did not show any increase in relative heart weight, it was increased in DT. In histology, DT showed cardiac fibrosis based on Masson/Goldner staining compared to WT. Basal ejection fraction was reduced in PP5-TG by 25%, by 29% in PP2C-TG, and by 38% of WT values in DT. This went hand in hand with a diminished phosphorylation of phospholamban at serine-16, the phosphorylation site for the cAMP-dependent protein kinase (PKA), after perfusion of isolated hearts with isoprenaline. DT and PP2C-TG sustained ischemia and reperfusion of isolated hearts worse than WT or PP5-TG. Expression of atrial natriuretic factor (ANF, marker of hypertrophy), of collagen 1a (fibrosis marker) and nuclear factor kappa B (marker of inflammation) in DT were augmented versus WT (ANF: 5.14 ± 0.75 vs. 1.00 ± 0.33; Col1a1: 1.82 ± 0.09 vs. 1.00 ± 0.10; Nfkb1: 1.33 ± 0.03 vs 1.00 ± 0.04; *n* = 6–10; *p* < 0.05). In summary, cardiac co-overexpression of two PPs, namely, PP2C and PP5, was detrimental to cardiac function. The underlying (patho)mechanism for this may involve dephosphorylation of Ca^2+^ regulatory proteins but also accompanying fibrosis and inflammation.

## Introduction

Serine and threonine phosphatases (PP) were detected and functionally active in heart muscle cells from mice but importantly also from man (Herzig and Neumann 2000, Neumann et al. 2021). There is evidence that the enzymatic activity of some isoforms of PP is elevated in some forms of human heart failure (review: Neumann et al. 2021, Fig. 1). Moreover, enhanced activity of, e.g., PP1 and/or PP2A, PP5, or PP2B was encountered in some animal models of cardiac hypertrophy and/or heart failure (Neumann et al. 1997). A pathological role of cardiac PP is further strengthened by the observation that cardiac specific overexpression of PP e.g. PP1, PP2A, PP2B, PP2C, or PP5 led to—time-dependent—cardiac hypertrophy and/or cardiac heart failure (Molkentin et al.^1998^; Gergs et al. 2004, 2012). In addition, one noted that some of these mice had genetic cardiac overexpression of PP but developed mechanisms for stress resistance (Morita et al. 2001, Hoehn et al. 2015; Gergs et al. 2019a). Therefore, cardiac PP can not only have deleterious but also beneficial roles for cardiac function. A better understanding of the underlying mechanisms may be important for the treatment of at least a subgroup of heart failure patients. As heart failure is an increasingly important cause of death in many countries, the present studies could be of clinical relevance.

**Fig. 1:**
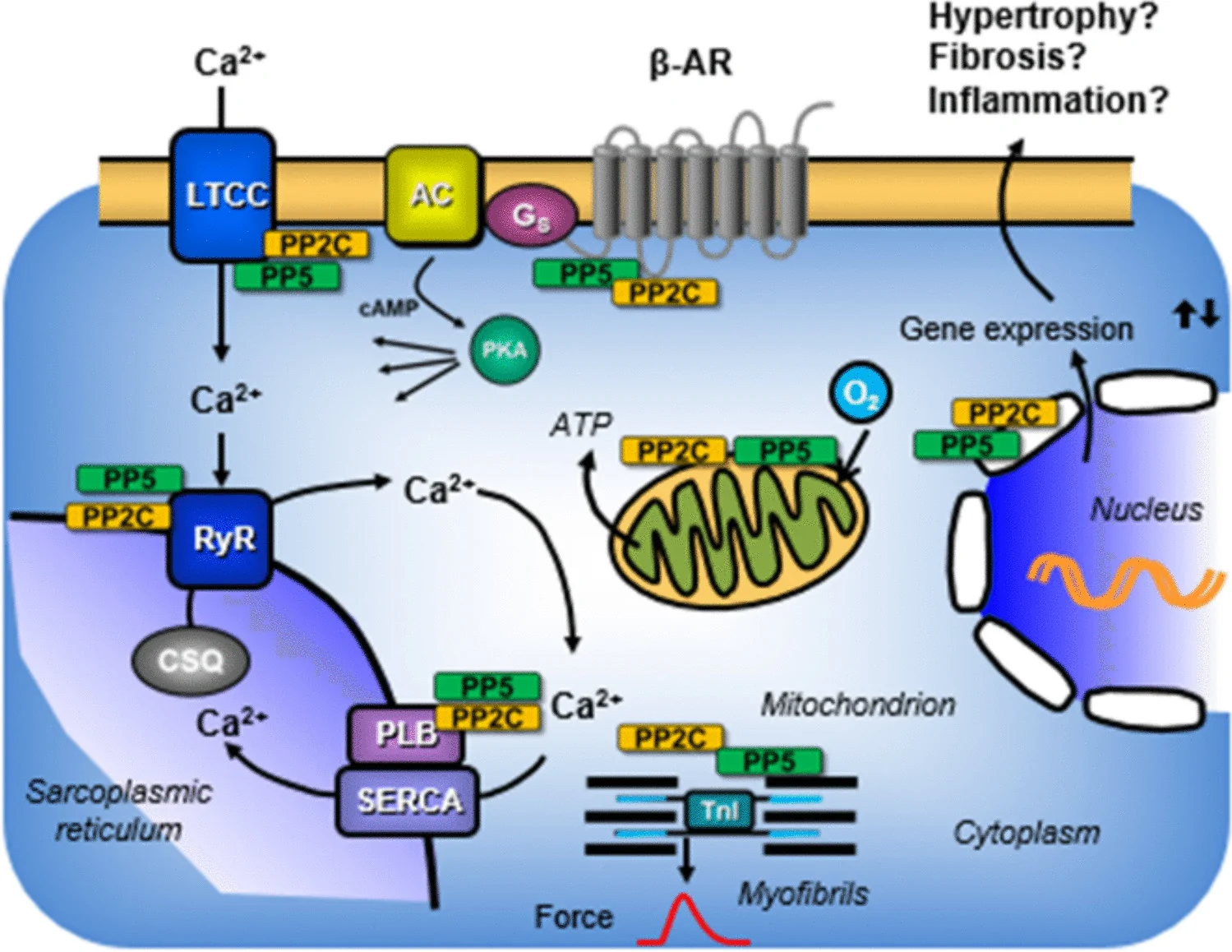
Scheme. β-Adrenergic stimulation augments the activity of adenylyl cyclases (AC) via stimulatory G-proteins (Gs). This increases cAMP-levels and cAMP activates cAMP-dependent protein kinases (PKA). PKA then phosphorylates regulatory proteins like phospholamban (PLB), the ryanodine receptor (RyR), the L-type Ca channel (LTCC), or troponin I (TnI) in the myofilaments. Finally, Ca^2+^ is elevated, binds to troponin C (TnC) which starts muscle contraction. These phosphorylations are in part reversed by protein phosphatases called PP2C or PP5. Additionally PP5 and PP2C are putative located in mitochondria and the nucleus of cardiomyocytes. Dephosphorylation of regulatory proteins in this compartments may influence cardiac apoptosis and gene expression

The phenotype of these mice with PP overexpression is often characterized by attenuated phosphorylation states of regulatory proteins which are located in different subcellular parts of cardiomyocytes (Fig. 1). These dephosphorylations of regulatory proteins may, in part, explain their phenotypes such as reduced force generation and prolonged tension relaxation. Based on these data, an inhibition of the enzymatic function of the various isoforms of PP with small organic molecules (tacrolimus for PP2C and okadaic acid for PP1) or peptides like inhibitor 1 or inhibitor-2 (Brüchert et al. 2008) might become useful to treat heart failure patients. In the present study, we address in part PP2C. We become interested in PP2C because it is expressed in all cells of mammals (Shi 2009, Chen et al. 2017). Thus, PP2C is also expressed in the human heart and in the mouse heart and here in cardiomyocytes. PP2C was first cloned in 1994 (Becker et al. 1994; Chen et al. 1994; Chinkers 1994, 1997). The expression of PP2C as mRNA was relatively high in the human heart compared to other organs of man (Chen et al. 1994). Limited data are available on the promoter of PP2C. It may be pathophysiologically relevant that there is a hypoxia response element in this promoter (Zhou et al. 2004, Wang et al. 2018). The other widely expressed protein that raised our attention is PP5. We had shown that selective cardiac overexpression of PP5 alone or PP2C alone in transgenic mice altered their cardiac function under basal conditions but also their resilience against stressors like increased catecholamine levels or ischemia and reperfusion (Gergs et al. 2012, 2019a; Bollmann et al. 2021).

In the present work, the effects of combined overexpression of two PPs, namely, PP2C and PP5, on cardiac performance, taking into account stress parameters such as β-adrenergic stimulation and oxidative stress, were investigated. Parts of the results have been published in abstract form (Schwarz et al. 2023a, b).

## Materials and methods

### Transgenic mice

We have described before that we generated transgenic mice which overexpressed the catalytic subunit of PP2C (PP2C) in the heart using a mouse cardiac α-myosin heavy chain promoter (Bollmann et al. 2021). We noted a 2.1 fold overexpression of PP2C on protein level in the hearts of these mice (Bollmann et al. 2021). Similarly, we have published the similarly generated mice with cardiac overexpression of PP5 (Gergs et al. 2012). In the present study we used six months old mice of each sex. The mice were kept under supervision in the animal facility (vivarium) of our Medical Faculty. The animal facility uses approved protocols of the animal welfare committee of the University of Halle-Wittenberg, Halle, Germany. Animal studies in our laboratory had approval of the Veterinäramt der Stadt Halle (Saale), Germany (approval reference number AZ 425–2–1518 MLU).

### Echocardiography

As described before (e.g. Bollmann et al. 2021; Dörner et al. 2021), we measured cardiac function in living mice. To that end, we performed transthoracic echocardiographic in spontaneously breathing mice. We used anesthesia with inhalation of 1.5% isoflurane. The echocardiography system was a Vevo 2100 system connected to a mouse-specific transducer (MS 550D transducer from Visual Sonics, Toronto, Canada). In the present study, we show results of two-dimensional images and M-mode recordings in the parasternal long axis view. In order to estimate the cardiac dimensions in our transgenic mice and in order to calculate the left ventricular ejection fraction from these original data, we utilized the Vevo®LAB software (Version 3.2.6 Vevo®2100, FUJIFILM VisualSonics®, Inc.).

### Langendorff perfusion

We measured force of contraction in the apex of isolated retrogradely, perfused spontaneously beating whole mouse heart following our published approaches (Gergs et al. 2004, 2010, 2019a, Dörner et al. 2021). The hearts were perfused with the help of a peristaltic pump at a constant flow rate of 2 ml per minute with a buffer. The buffer had the following composition (in millimolar): NaCl, 119.8; KCl, 5.4; CaCl_2_, 1.8; MgCl_2_, 1.05; NaH_2_PO_4_, 0.42; NaHCO_3_, 22.6; Na_2_EDTA, 0.05; ascorbic acid, 0.28; and glucose, 5.05. This buffer was continuously gassed with 95% O_2_ and 5% CO_2_ in a beaker and kept at 37 °C (Neumann et al. 2003; Kirchhefer et al. 2004). Developed force was taken from the apex cordis by a metal hook connected to an electronic force monitor. The signals were fed into a bridge amplifier. They were digitized and then analyzed by means of the commercial software Labchart (AD Instruments, Oxford, England). Perfusion of hearts with drugs took place though a side-arm with a syringe connected to a pump. This pump was connected as a bypass with the aorta. At the end of experiments, hearts were shock frozen (using aluminum tongues, precooled in liquid nitrogen) in liquid nitrogen: this was done to freeze any phosphorylation or in the perfused hearts reactions. Frozen whole heart samples were maintained kept at − 80 °C and then thawed for Western blotting.

### Preparation of homogenates

Frozen hearts were pulverized in a still frozen state (Mikro-Dismembrator, Sartorius, Göttingen, Germany). The dismembrator used plastic tubes and wolfram beads precooled in liquid nitrogen. To the frozen heart powder, sitting in liquid nitrogen, we added 100 µl of a solvent that was comprised of 10 mM NaHCO_3_ and 5% SDS. This solvent was used to block any proteolysis and dephosphorylation of cardiac proteins in the heart samples. We then homogenized tissue and solvent with a Sonopuls ultrasonic homogenizer (Bandelin, Berlin, Germany). These steps were carried out on ice. Then, crude extracts were incubated at room temperature for 30 min in order to let it thaw. Then, we centrifuged the samples (30 min, 14.000 × *g*) to remove a pellet. The supernatants (called “homogenates”) were separated from the pellet and stored at − 80 °C until further use. Proteins in these homogenates were quantified by means of the Lowry protein assay (Lowry et al. 1951).

### Western blot analysis

We performed the electrophoresis, the incubation with antibodies, and the quantification of the antibody signals as previously described (Gergs et al. 2009, Boknik et al. 2018; Gergs et al. 2019a). We used for the electrophoresis of the cardiac samples precast gradient gels, namely, Novex™ 4–20% “Tris–Glycine Plus Midi Protein Gels” (Invitrogen, Thermo Fisher Scientific, Waltham, Massachusetts, USA). We ran the gels at 4 °C for about one hour at 120 V in the a commercial buffer, called “NuPAGE MES SDS Running Buffer” (Thermo Fisher Scientific, Waltham, Massachusetts, USA) using an XCell4 SureLock™ Midi-Cell chamber (Life Technologies by Thermo Fisher Scientific, Waltham, Massachusetts, USA). After the run of the gel, the proteins were transferred to nitrocellulose membranes (Amersham™ Protran, GE Healthcare, Chicago, Illinois, USA) at 4 °C. We blocked the nitrocellulose membranes for one hour at room temperature. Thereafter, we incubated the cut membranes overnight in a cold room (4 °C) with the antibody against serine 16-phosphorylated phospholamban (dilution was 1:5000; product number: A010-12AP; Badrilla, Leeds, England), against total phospholamban (that is against phosphorylated and not phosphorylated phospholamban, diluted 1:7500; product number: A010-14; Badrilla, Leeds, England), against PP2C (dilution 1:500; product number: sc-166662; Santa Cruz Biotechnology, Dallas, United States of America) and PP5 (1:2000; catalog number: 611021; BD Biosciences, New Jersey, United States of America). We used an antibody against calsequestrin (1:20.000; product number: ab3516; abcam, Cambridge, England) as a loading control between the lanes of the gels. The antibody against SERCA (dilution 1:1000; product number: ab2861; abcam, Cambridge, UK) was used. We visualized the signals due to the secondary antibodies with the help of the chemiluminescent HRP substrate (Immobilon™ Western, Milli pore, Merck; Darmstadt, Germany). The luminescence was quantified with a commercial digital imaging system (Amersham ImageQuant 800; Cytiva Europe GmbH, Freiburg im Breisgau, Germany).

### Histological analysis

The preparation of cardiac apex samples, mounting, and staining were carried out as published before (Gergs et al. 2004). To analyze the morphological features of transgenic myocardium, dewaxed and rehydrated tissue sections of formaldehyde-fixed probes were stained as follows: hematoxylin and eosin (HE) to assess cell size and nuclei, with Masson–Goldner trichrome (MG) to assess fibrosis in the mouse hearts. We sued for imaging a Zeiss Axio Imager Z1 microscope with an AxioCam Digital microscope camera. We analyzed the slices with a commercial software (AxioVision from Carl Zeiss Vision GmbH, Aalen, Germany).

### Real-time PCR

We isolated total RNA from frozen mouse hearts with the help of a commercial kit: the TRIzol reagent from Invitrogen (Fisher Scientific, Schwerte, Germany) following the instructions of the manufacturer. Thereafter, we performed reverse transcription of the RNA by means of another kit: the high-capacity cDNA Reverse Transcription Kit with RNAse inhibitors from Applied Biosystems (Grand Island, New York, United States of America) following the instructions of the manufacturer. We performed the reverse transcription with 200 ng/μl RNA. Then, we diluted the cDNA samples to a volume corresponding to 2 ng RNA per μl. We achieved the real-time PCR amplification and detection by means of the commercial QuantStudio™ 3 Real-Time PCR System from Thermo Fisher Scientific (Schwerte, Germany) using SYBR™ Select Master Mix (Thermo Fisher Scientific) following the instruction of the manufacturer. The relative expression of the genes of interest was calculated in LinReg software (https://medischebiologie.nl/files/), and the resulting expression was calculated according to Pfaffl method (Pfaffl 2001) using the Hprt1 signal for normalization. We developed the primers for the amplification with the software Primer-BLAST (Ye et al. 2012). We defined genes with Cq values higher than 35 (Cq > 35) as not expressed in the mouse heart. The forward and reverse primer sequences specific for each gene of interest are listed as follows: ANF (encoded by Nppa gene): CGTCTTGGCCTTTTGGCTTC, GGTGGTCTAGCAGGTTCTTGAAA; Col1a1: CCCTGGTCCCTCTGGAAATG, GGACCTTTGCCCCCTTCTTT; Nfkb (encoded by Nfkb1 gene): AGAGCAACCAAAACAGAGGGG, GTTGCAAATTTTGACCTGTGGG; Il6: CCGGAGAGGAGACTTCACAG, TTCTGCAAGTGCATCATCGT and reference gene Hprt1: TTGGGCTTACCTCACTGCTT, ATCATCGCTAATCACGACGC. To test the expression of the transgenes in the heart of transgenic mice, we assessed expression of endogenous and exogenous PP5 and PP2C. For this purpose, we designed the primer sequences with respect to the construct sequences of both transgenes. The used primer pair sequences are listed here: exogenous PP5: ACAACGCTGAAAGAGACAGAGA, GGTCCACAAAGTCGCCGTTA; endogenous PP5 (encoded by Ppp5c gene): ACTCGGCACTCCTTCGC, CAAAGCGACCCCCACCC; exogenous PP2C: AGGTCTCAGATGAAGCAATGAGA, CAGACAAGATGCGCATGACG and endogenous PP2C (encoded by Ppm1b gene): GCTGCTCAATGGCGGAAAAG, CGGACACGGAGAAACAGAGA).

### Data analysis

For reasons of comparability with previous studies (e.g., Bollmann et al. 2021), we present data as arithmetic means ± S.E.M. We tested for statistical significance with the help of analysis of variance. Thereafter we used Bonferroni’s *t*-test or Student’s *t*-test. We defined a *p*-value smaller or equal to 0.05 as significant. We used GraphPad Prism 9 for plotting experimental data such as agonist-induced positive inotropic and chronotropic effects. We achieved statistical analyses as indicated in the figures and tables. We conducted statistical evaluation with GraphPad Prism 9 (GraphPad Software, San Diego, California, United States of America).

### Drugs and materials

We obtained (−)-isoprenaline (+)-bitartrate from Sigma-Aldrich (Deisenhofen, Germany). All other chemicals were of the highest purity grade that we could buy on the market. Deionized water was used for the buffers and other solution in our experiments. Stock solutions were prepared daily.

## Results

**Fig. 2:**
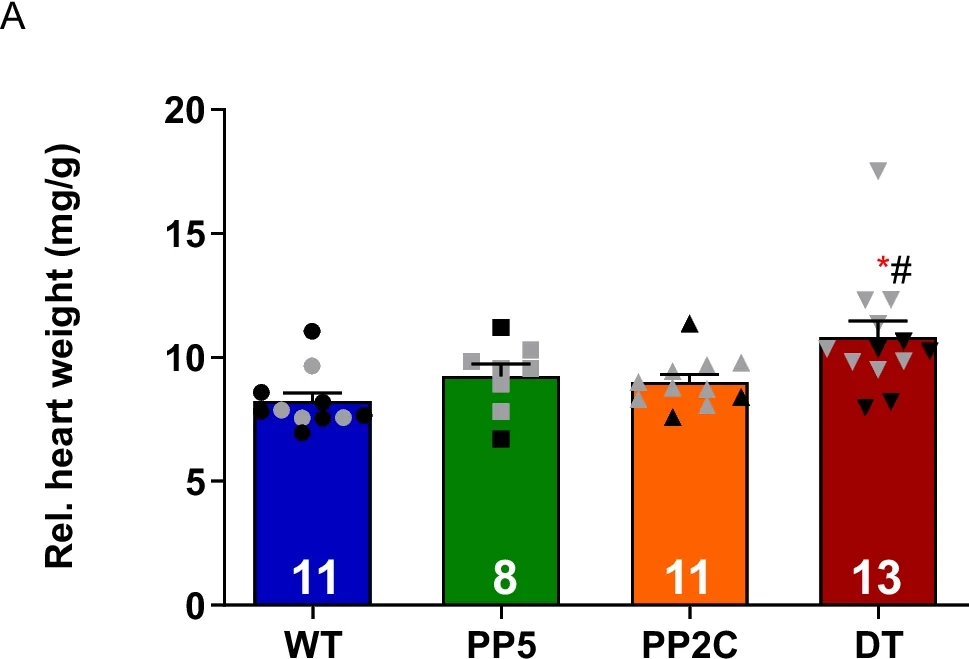
Cardiac gravimetry. **A** Bar diagram of the relative heart weight of WT, PP5-TG, PP2C-TG, and DT mice. ^*^p < 0.05 vs. WT and ^#^p < 0.05 vs. PP2C. Significances calculated with one-way ANOVA (red), with *t*-test (black). Number in bars indicate number of studied animals. Black individual symbols indicate female animals and gray indicate male animals

**Fig. 3:**
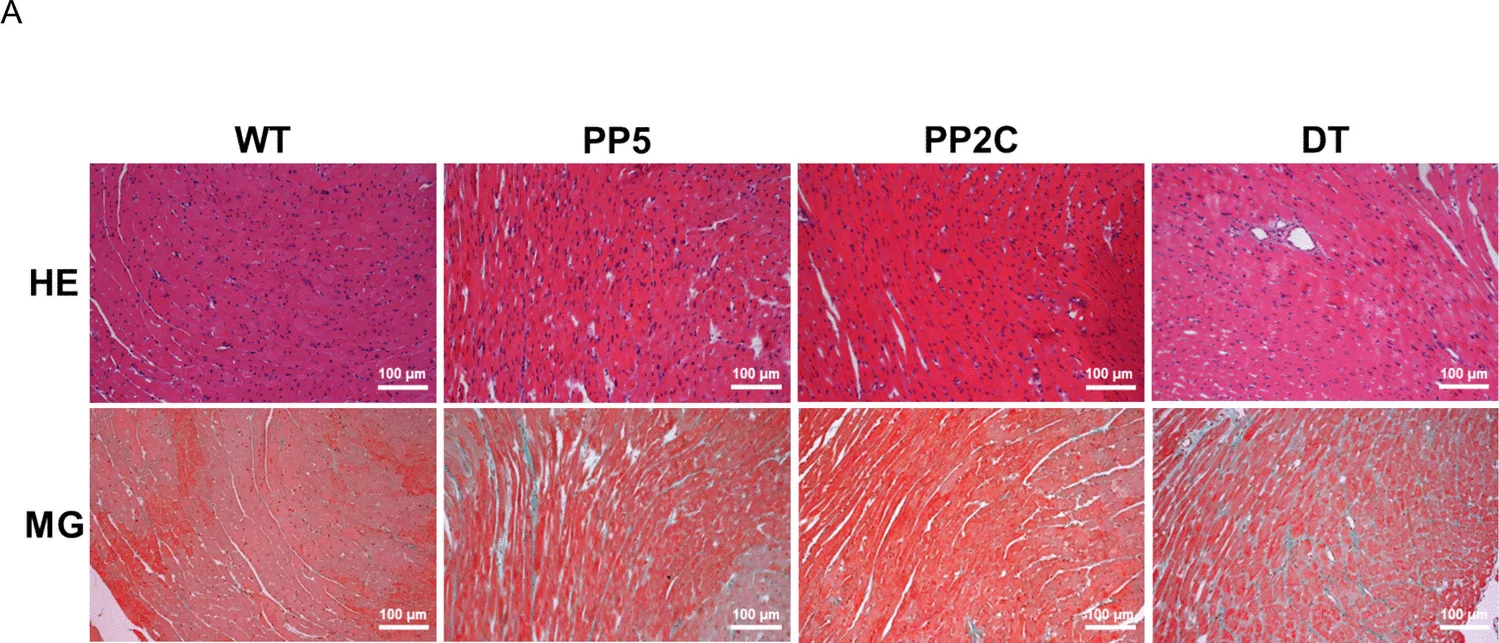
Histological staining. **A** Hematoxylin–eosin staining (top) and Masson–Goldner staining (bottom) of WT, PP5-TG, PP2C-TG, and DT hearts

As depicted in Fig. 2A, mean relative heart weight was higher in PP2C-TG and PP5-TG mice than in WT (but not significantly). In contrast, DT had a significantly larger relative heart weight than WT or PP2C. Histologically, no gross alterations between phenotypes were noted in HE staining; however, in Masson–Goldner staining a slight fibrosis in DT was notable (Fig. 3A).

**Fig. 4:**
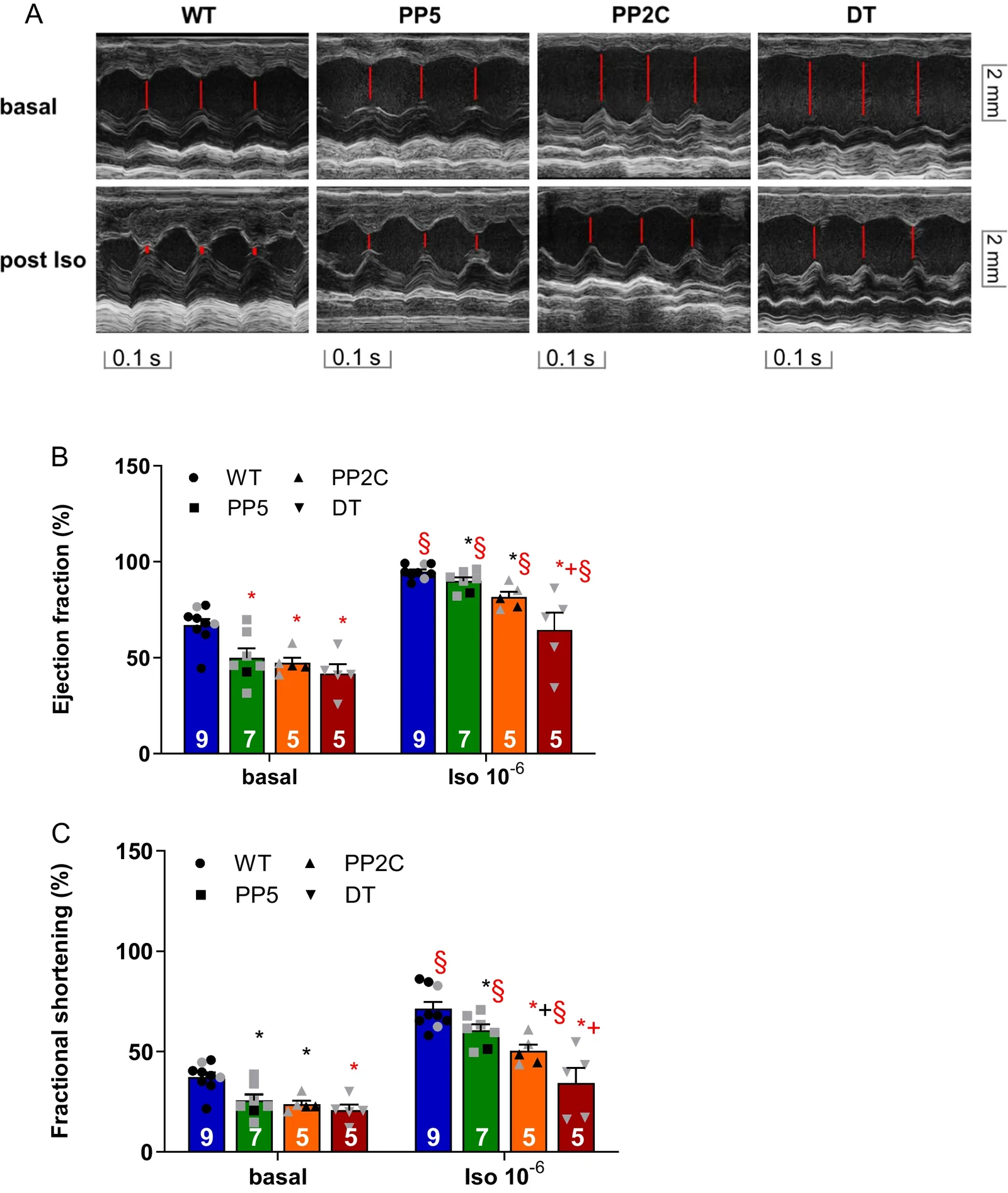

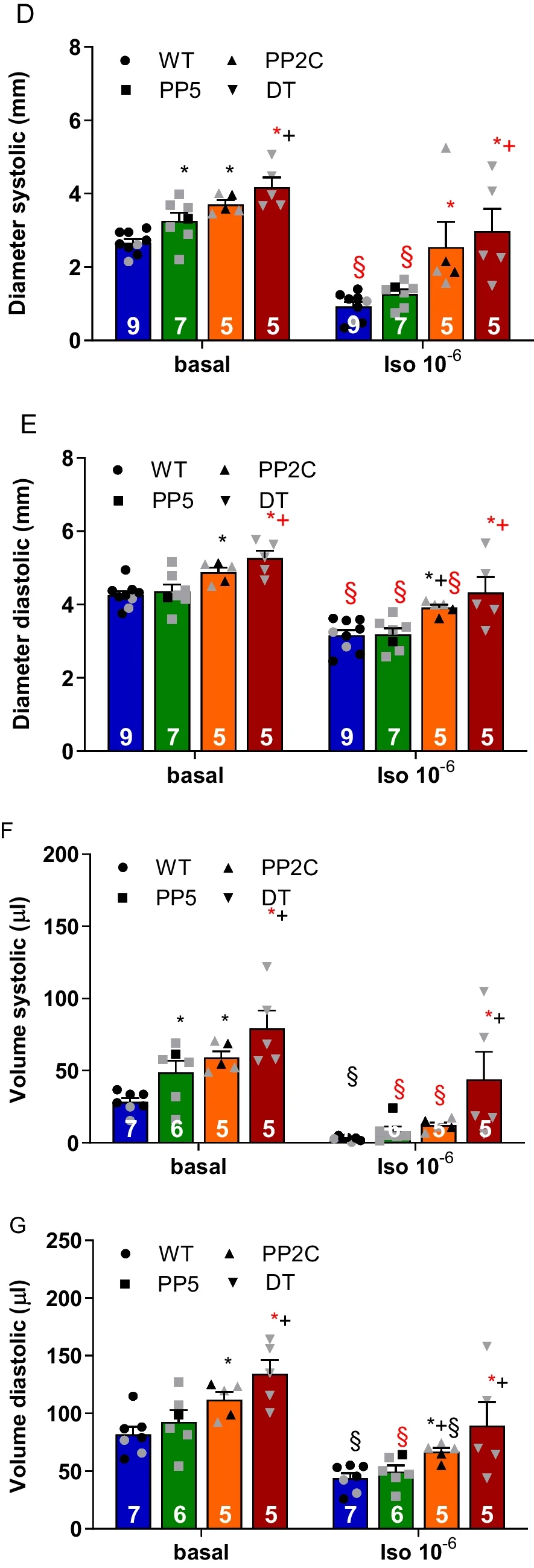
Echocardiography. **A** Original images of the m-mode in the long axis view of WT, PP5-TG, PP2C-TG, and DT hearts basal (top) and after β-adrenergic stimulation with 1 µM isoprenaline (bottom). Left ventricular diameter (mm, ordinate) over time (ms, abscissa). **B** Bar diagram of the ejection fraction (output of the left ventricle during contraction) in % of WT, PP5-TG, PP2C-TG, and DT mouse basal and after β-adrenergic stimulation with 1 µM isoprenaline. ^*^p < 0.05 vs. WT, ^+^*p* < 0.05 vs. PP5, and ^§^*p* < 0.05 vs. basal. Significances calculated with one-way ANOVA (red), with *t*-test (black). Number in bars indicate number of studied animals. Black individual symbols indicate female animals, grey indicate male animals. **C** Bar diagram of the fractional shortening (size reduction of the left ventricle during systole) in % of WT, PP5-TG, PP2C-TG, and DT mouse basal and after β-adrenergic stimulation with 1 µM isoprenaline. ^*^p < 0.05 vs. WT, ^+^*p* < 0.05 vs. PP5, and ^§^*p* < 0.05 vs. basal. Significances calculated with one-way ANOVA (red), with *t*-test (black). Number in bars indicate number of studied animals. Black individual symbols indicate female animals, grey indicate male animals. **D** Bar diagram of the end systolic diameter of the left ventricle in mm of all four genotypes basal and after 1 µM isoprenaline. ^*^p < 0.05 vs. WT, ^+^*p* < 0.05 vs. PP5, and ^§^*p* < 0.05 vs. basal. Significances calculated with one-way ANOVA (red), with *t*-test (black). Number in bars indicate number of studied animals. Black individual symbols indicate female animals, grey indicate male animals.  **E** Bar diagram of the end-diastolic diameter of the left ventricle in mm of WT, PP5-TG, PP2C-TG, and DT mouse basal and after β-adrenergic stimulation. ^*^p < 0.05 vs. WT, ^+^*p* < 0.05 vs. PP5, and ^§^*p* < 0.05 vs. basal. Significances calculated with one-way ANOVA (red), with *t*-test (black). Number in bars indicate number of studied animals. Black individual symbols indicate female animals, gray indicate male animals. **F** Bar diagram of the end-systolic volume of the left ventricle in µl of all four genotypes basal and after β-adrenergic stimulation. ^*^p < 0.05 vs. WT, ^+^*p* < 0.05 vs. PP5, and ^§^*p* < 0.05 vs. basal. Significances calculated with one-way ANOVA (red), with *t*-test (black). Number of bars indicate number of studied animals. Black individual symbols indicate female animals, and gray indicate male animals. **G** Bar diagram of the end-diastolic volume of the left ventricle in µl of WT- PP5-TG, PP2C-TG, and DT mouse basal and after 1 µM isoprenaline. ^*^p < 0.05 vs. WT, ^+^*p* < 0.05 vs. PP5, and ^§^*p* < 0.05 vs. basal. Significances calculated with one-way ANOVA (red), with *t*-test (black). Number in bars indicate number of studied animals. Black individual symbols indicate female animals and gray indicate male animals

We noted an impairment of basal left ventricular ejection fraction (EF) in DT compared to the WT. This is denoted in an original recording of an M-mode echocardiography (Fig. 4A, top row). Data for the EF were quantified and were summarized in Fig. 4B (left hand side). Of note, EF was lower in PP2C-TG, PP5-TG, and DT in comparison to WT. One knows that impairments in left ventricular performance might be seen better after β-adrenergic stimulation. Hence, we injected all four genotypes of mice with isoprenaline, a β-adrenergic agonist and recorded again EF. Here, we detected in all genotypes an increase in the ejection fraction compared to basal line values. This indicates that all mice are responsive to β-adrenergic stimulation, as we expected (Fig. 4B left hand side compared to right hand side). Moreover, the EF after acute injection of isoprenaline was the smallest in DT followed by PP2C-TG and PP5-TG. This confirms the impaired function of DT to sustain this kind of stress (Fig. 4B right hand side) and proves that stress (due to isoprenaline on β-adrenoceptors) made differences in EF between the tested genotypes more visible. Fractional shortening is another clinically often used parameter to assess EF. However, the same pattern as for EF itself was noted with fractional shortening. Fractional shortening was reduced in all transgenic lines compared to WT under basal conditions (Fig. 4C left hand side). The fractional shortening was enhanced after acute injection with isoprenaline in all mouse lines (Fig. 4C right hand side). As in Fig. 4B, fractional shortening was lowest in DT under isoprenaline compared to the other genotypes investigated (Fig. 4C). Next, we assessed the end systolic diameter under these conditions (Fig. 4D). This diameter under basal conditions was highest in DT and notably higher than in WT (Fig. 4D, left hand side). When the contractility was enhanced by injection of isoprenaline, then, the end systolic diameter (a measurement how well the left ventricle is able to empty itself from blood and increase cardiac output into the aorta and thus into the peripheral arterial vessels) was reduced in all genotypes but the diameter remained higher in DT than in WT. This indicates that the systolic function is increasingly impaired in DT. These changes were similar in the end diastolic function (Fig. 4E). This is a measurement of the relaxation of the left ventricle. The end systolic volume under basal conditions was higher in DT than in WT (Fig. 4F, left). Under isoprenaline injection the end systolic volume was highest in DT (Fig. 4F, right). Under basal conditions, systolic volume was largest in DT and was reduced by isoprenaline in all genotypes. However, isoprenaline reduced systolic volume much less in DT than in WT (Fig. 4F). Under basal conditions, diastolic volume was largest in DT and was reduced by isoprenaline in all genotypes. However, isoprenaline increased diastolic volume much less in DT than in WT (Fig. 4G).

**Fig. 5:**
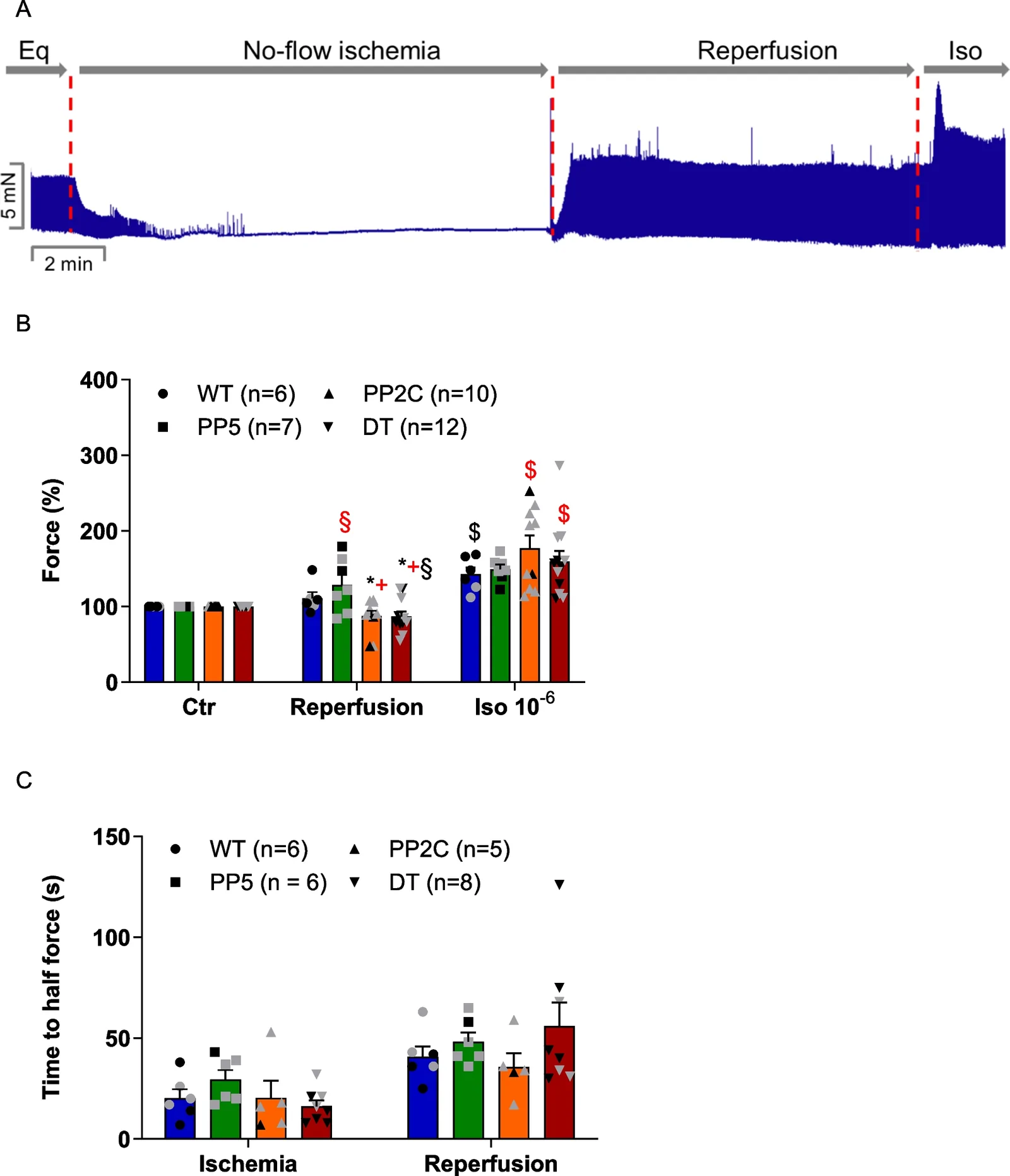

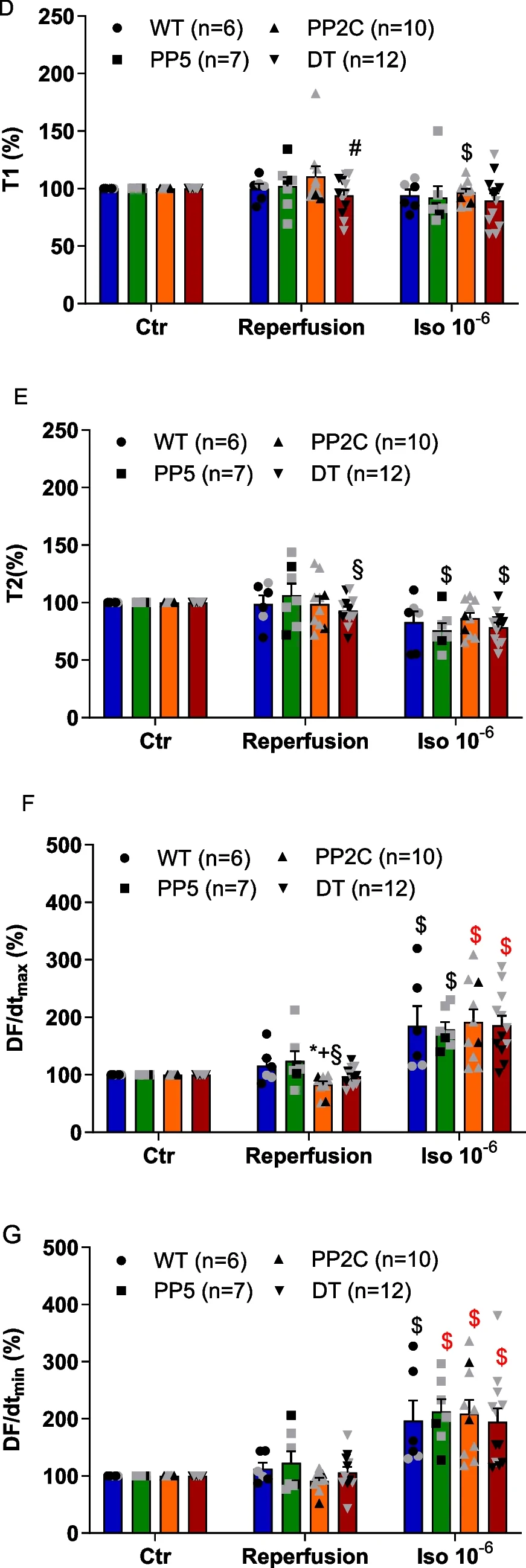
Langendorff perfusion. **A** Original recording of the time course of equilibration (Eq, 30 min), force reduction during no-flow ischemia (20 min), force recovery during reperfusion (10 min), and the effect of β-adrenergic stimulation with 1 µM isoprenaline (Iso) in isolated perfused heart preparations. Force of contraction in milli Newton (mN, ordinate) over time in minutes (min, abscissa). **B** Bar diagram of force of contraction in % of WT, PP5-TG, PP2C-TG and DT mouse basal (Ctr = 100%), after reperfusion and after β-adrenergic stimulation. ^*^p < 0.05 vs. WT, ^+^*p* < 0.05 vs. PP5, ^§^*p* < 0.05 vs. Ctr, and ^$^*p* < 0.05 vs. reperfusion. Significances calculated with one-way ANOVA (red), with *t*-test (black). *n* indicates number of studied animals. Black individual symbols indicate female animals and gray indicate male animals. **C** Bar diagram of time to half force in seconds during ischemia and reperfusion of all four genotypes. *n* indicates number of studied animals. Black individual symbols indicate female animals and gray indicate male animals. **D** Bar diagram of time to peak tension (t1) in % of all four genotypes basal (Ctr = 100%), after reperfusion and β-adrenergic stimulation. ^#^*p* < 0.05 vs. PP2C, ^$^*p* < 0.05 vs. reperfusion, calculated with *t*-test (black). *n* indicates number of studied animals. Black individual symbols indicate female animals and gray indicate male animals. **E** Bar diagram of time to relaxation (t2) in % of all four genotypes basal (Ctr = 100%), after reperfusion and β-adrenergic stimulation. ^§^*p* < 0.05 vs. Ctr and ^$^*p* < 0.05 vs. reperfusion, calculated with t-test (black). n indicates number of studied animals. Black individual symbols indicate female animals and gray indicate male animals. **F** Bar diagram of rate of tension development (dF/dt_max_) in % of WT, PP5-TG, PP2C-TG, and DT mouse basal (Ctr = 100%), after reperfusion and treatment with 1 µM isoprenaline. ^*^p < 0.05 vs. WT, ^+^*p* < 0.05 vs. PP5, ^§^*p* < 0.05 vs. Ctr, and ^$^*p* < 0.05 vs. reperfusion. Significances calculated with One-Way ANOVA (red), with t-test (black). n indicates number of studied animals. Black individual symbols indicate female animals, grey indicate male animals. **G** Bar diagram of rate of tension relaxation (dF/dt_min_) in % of WT, PP5-TG, PP2C-TG, and DT mouse basal (Ctr = 100%), after reperfusion and treatment with 1 µM isoprenaline. ^$^*p* < 0.05 vs. reperfusion. Significances calculated with one-way ANOVA (red), with *t*-test (black). *n* indicates number of studied animals. Black individual symbols indicate female animals and gray indicate male animals

As a next step, we isolated hearts and perfused them in the Langendorff mode. This is depicted in the original recording Fig. 5A. During no-flow, ischemia force rapidly declined. When perfusion was reinstated force increased again. Finally, 1 µM isoprenaline was added to stimulate force of contraction maximally (Fig. 5A). In perfused isolated hearts, basal force was set as 100% in all genotypes (Fig. 5B). Then, ischemia and reperfusion was performed in all genotypes. After reperfusion, in DT, less force was generated than in WT. Thereafter, isoprenaline was added. This increased force of contraction. Here, the increase was less in DT than in WT (Fig. 5B). The time to half-maximal restitution of force was unchanged between all groups under ischemia and under subsequent reperfusion (Fig. 5C). Isoprenaline increased force of contraction in WT, PP2C, and DT (Fig. 5B). However, the isoprenaline-induced increase in force of contraction was similar in DT and in PP2C-TG (Fig. 5B). Isoprenaline reduced time to peak tension only in PP5 and DT (Fig. 5D) while reperfusion shortened time of relaxation in DT (Fig. 5E). Interestingly, when we applied isoprenaline, the rate of tension development increased in all genotypes (Fig. 5F). Isoprenaline increased the rate of relaxation in all genotypes (Fig. 5G). This parameter was not significantly affected by reperfusion (Fig. 5G).

**Fig. 6:**
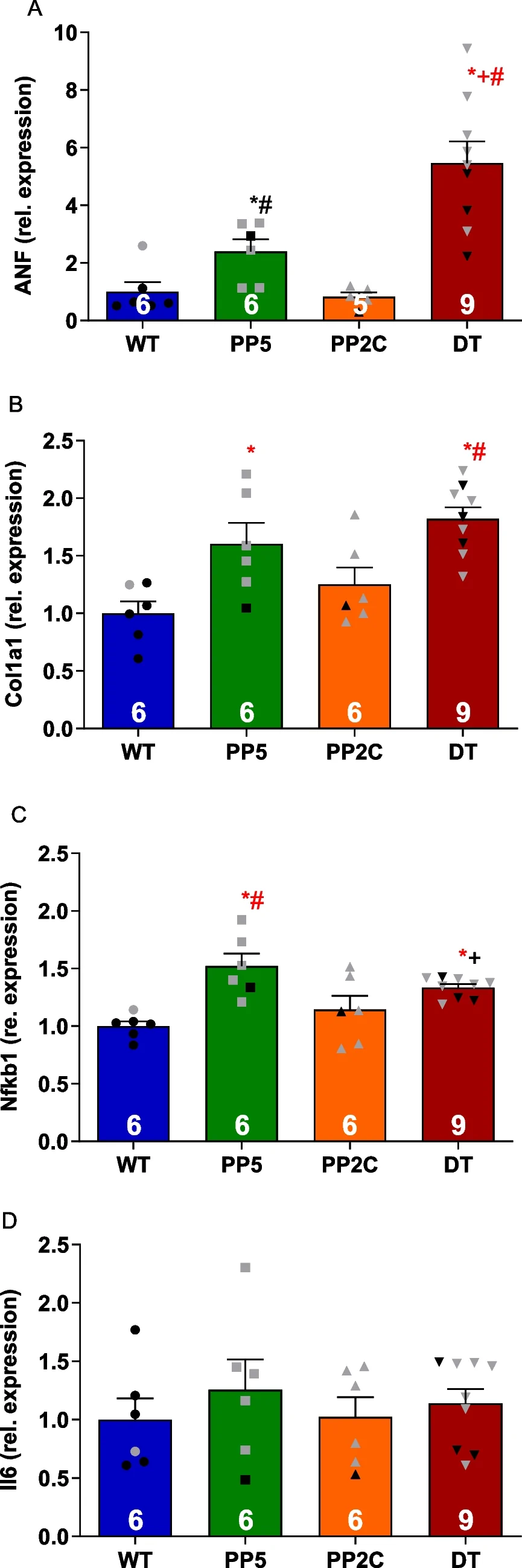
mRNA quantification. **A** Bar diagram of the relative expression of the atrial natriuretic factor (ANF) of WT, PP5-TG, PP2C-TG, and DT mice. ^*^p < 0.05 vs. WT, ^+^*p* < 0.05 vs. PP5, and ^#^*p* < 0.05 vs. PP2C. Significances calculated with one-way ANOVA (red), with *t*-test (black). Number in bars indicates number of studied animals. Black individual symbols indicate female animals, grey indicate male animals. **B** Bar diagram of the relative expression of the collagen, type I, alpha 1 (Col1a1) of WT, PP5-TG, PP2C-TG, and DT mice. ^*^p < 0.05 vs. WT and ^#^*p* < 0.05 vs. PP2C. Significances calculated with ONE-WAY ANOVA (red). Number in bars indicates number of studied animals. Black individual symbols indicate female animals and gray indicate male animals. **C** Bar diagram of the relative expression of the nuclear factor Nfkb1 of all four genotypes. ^*^p < 0.05 vs. WT, ^+^*p* < 0.05 vs. PP5, and ^#^*p* < 0.05 vs. PP2C. Significances calculated with one-way ANOVA (red), with *t*-test (black). Number in bars indicates number of studied animals. Black individual symbols indicate female animals and gray indicate male animals. **D** Bar diagram of the relative expression of interleukin 6 (IL 6) of all four genotypes. Number in bars indicates number of studied animals. Black individual symbols indicate female animals and gray indicate male animals

Next, we isolated RNA from all genotypes. We quantified mRNA of genes of interest. We noted that the mRNA for ANF (a parameter for heart failure and hypertrophy) was elevated in PP5-TG and DT but not PP2C in comparison to WT (Fig. 6A). Histologically, we noted fibrosis in DT compared to WT (Fig. 2B). The mRNA of the fibrosis marker Col1a1 was increased not only in DT but also PP5-TG in comparison to WT (Fig. 6B). Moreover, a marker of inflammation (Nfkb) was increased in DT and PP5-TG in comparison to WT (Fig. 6C). This increase was larger in PP5-TG than in DT and might indicate specificity. In contrast, the mRNA for interleukin 6 was unaltered between genotypes (Fig. 6D).

**Fig. 7:**
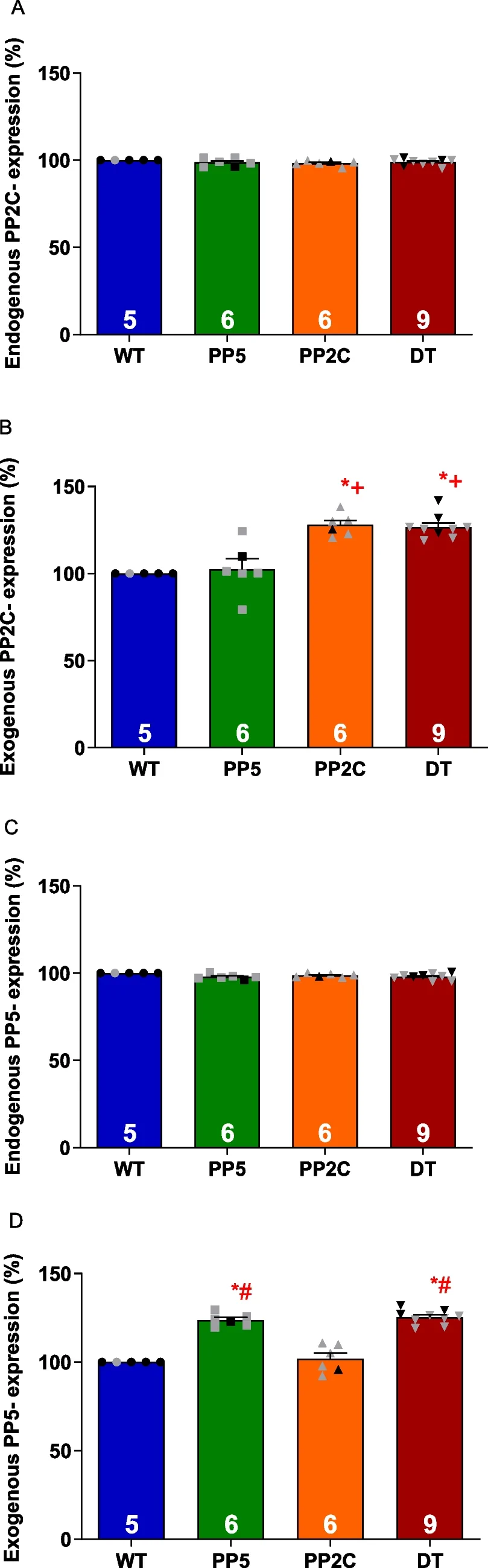
Overexpression of PP2C and PP5. **A** Bar diagram of the endogenous mRNA level of PP2C in WT, PP5-TG, PP2C-TG, and DT mice. Number in bars indicates number of studied animals. Black individual symbols indicate female animals and gray indicate male animals. **B** Bar diagram of the exogenous mRNA level of PP2C in WT, PP5-TG, PP2C-TG, and DT mice. ^*^p < 0.05 vs. WT and ^+^*p* < 0.05 vs. PP5, calculated with one-way ANOVA (red). Number in bars indicate number of studied animals. Black individual symbols indicate female animals and gray indicate male animals. **C** Bar diagram of the endogenous mRNA level of PP5 in WT, PP5-TG, PP2C-TG, and DT mice. Number in bars indicates number of studied animals. Black individual symbols indicate female animals and gray indicate male animals. **D** Bar diagram of the exogenous mRNA level of PP5 in WT, PP5-TG, PP2C-TG, and DT mice. ^*^p < 0.05 vs. WT and ^#^*p* < 0.05 vs. PP2C, calculated with one-way ANOVA (red). Number in bars indicate number of studied animals. Black individual symbols indicate female animals and gray indicate male animals

The mRNA level for endogenous PP2C (Fig. 7A) and PP5 (Fig. 7C) was unaltered between genotypes. The mRNA for the exogenous PP2C was higher in PP2C-TG and DT compared to WT and PP5-TG mice (Fig. 7B, as expected). Likewise, the mRNA for the exogenous PP5 was higher in PP5-TG and DT compared to WT and PP2C-TG (Fig. 7D).

**Fig. 8:**
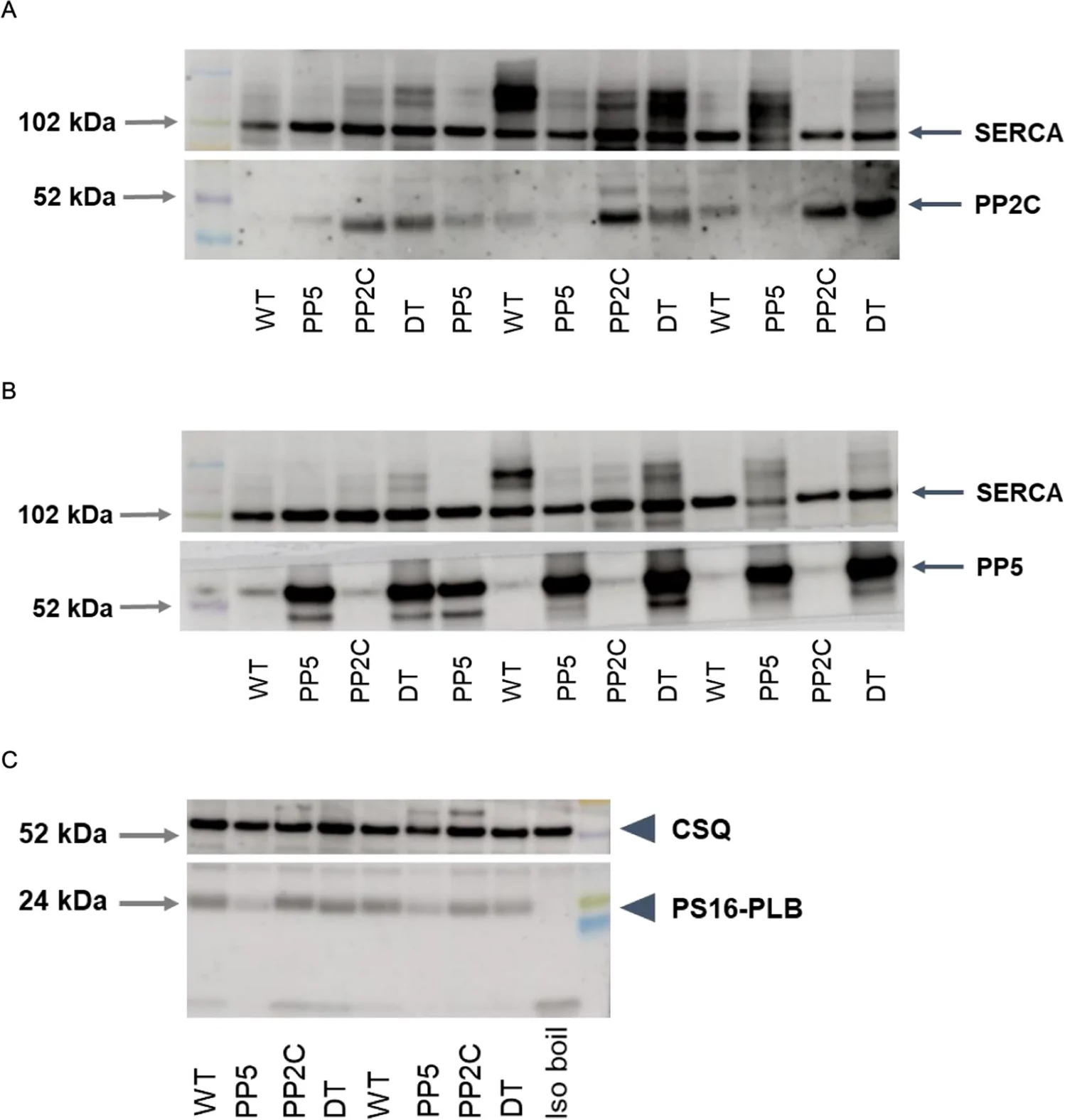

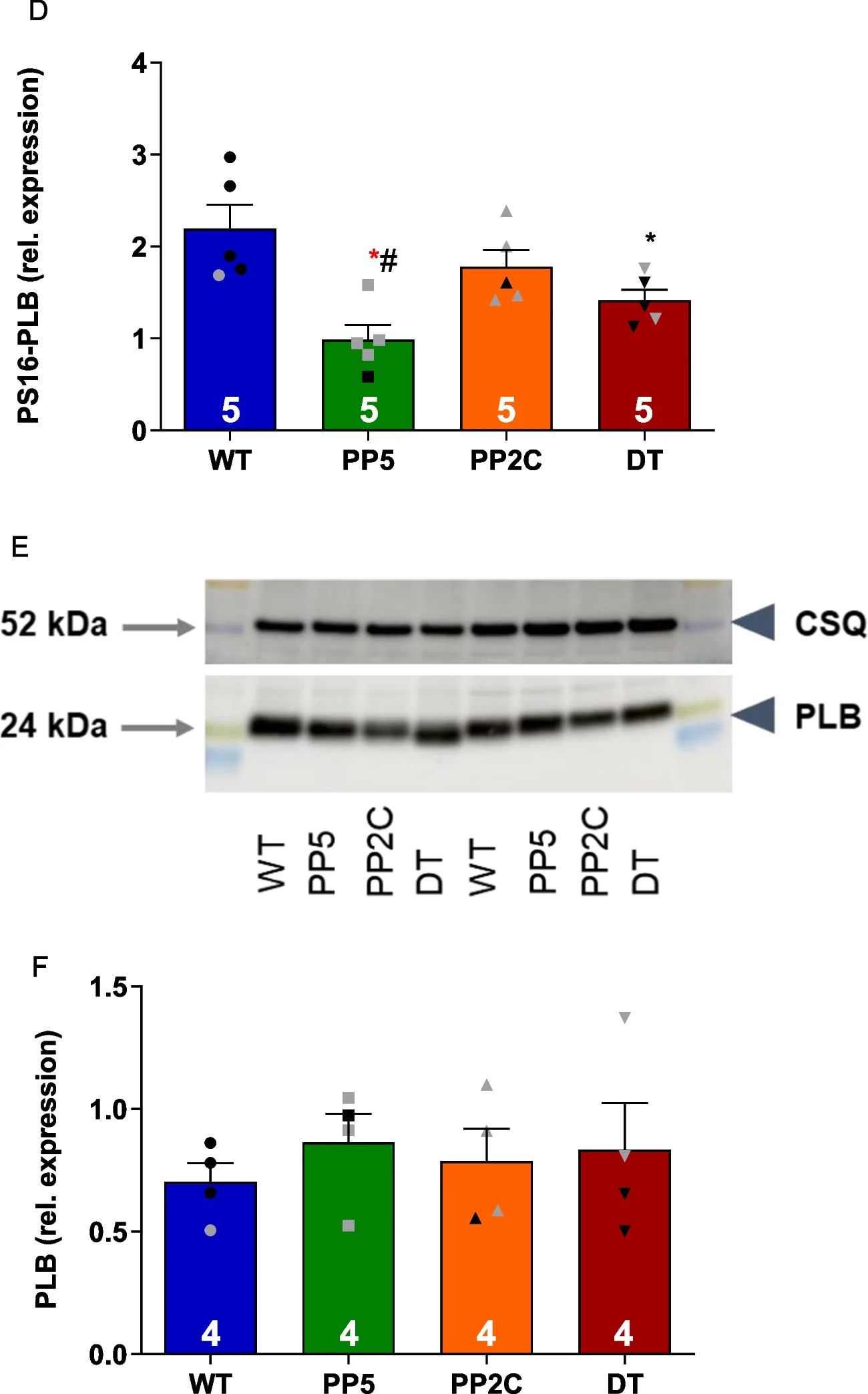
Western blotting. **A** Original western blot detecting for PP2C in all four genotypes, with SERCA as loading control. **B** Original western blot detecting for PP5 in all four genotypes, with SERCA as loading control.  **C** Original western blot detecting for phosphorylated PLB for WT, PP5-TG, PP2C-TG, and DT mice, with calsequestrin (CSQ) as loading control. Identity of PLB was confirmed by running boiled and unboiled samples. Boiling confirms the conversion of the high molecular weight form of PLB to a low molecular weight form. This change is typical for PLB. **D** Bar diagram for the expression of phosphorylated PLB calculated relative to calsequestrin (CSQ) for WT, PP5-TG, PP2C-TG, and DT mice. ^*^p < 0.05 vs. WT and ^#^*p* < 0.05 vs. PP2C. Significances calculated with one-way ANOVA (red), with *t*-test (black). Number in bars indicate number of studied animals. Black individual symbols indicate female animals and gray indicate male animals. **E** Original western blot detecting total PLB for WT, PP5-TG, PP2C-TG, and DT mice, with calsequestrin (CSQ) as loading control. **F** Bar diagram for expression of total PLB calculated relative to CSQ for WT, PP5-TG, PP2C-TG, and DT mice. Number in bars indicates number of studied animals. Black individual symbols indicate female animals and gray indicate male animals

At the end of the perfusion (Fig. 5A), the heart was freeze-clamped in liquid nitrogen. From these frozen samples, homogenates were prepared and subjected to Western blotting. First, it was of interest to assess the level of protein overexpression of PP. As seen in original experiments, the patterns of overexpression were as expected. PP2C as protein was overexpressed in PP2C-TG and DT compared to WT and PP5-TG (Fig. 8A). In difference, PP5 was overexpressed in PP5-TG and DT compared to WT and PP2C-TG (Fig. 8B).

As a functional read out for PP activity, we measured in these samples the phosphorylation state of serine-16 in phospholamban (Fig. 8C). Here, we noted that in DT less phosphorylation was measured than in WT. Of note, phosphorylation was even less in PP5-TG (Fig. 8C). These data were quantified in Fig. 8D.

## Discussion

We have shown before that when we overexpressed PP2C in the heart of transgenic mice, cardiac hypertrophy ensues (Bollmann et al. 2021; Dörner et al. 2021). We noted a similar cardiac hypertrophy but less strong in mice that overexpressed the PP5 in the cardiomyocytes (Gergs et al. 2012). Here, we tested the hypothesis that the effects of the PP5 can influence the effects of the PP2C. This was not unlikely because PP in general are not very substrate specific. There was an overlap between the PP that are dephosphorylated by PP5-TG and PP2C (Neumann et al. 2021).

Indeed, we noted a much deteriorated phenotype in DT mice. One might call this an additive effect. More specifically, the heart weight increased substantially in DT. Histological data indicate fibrosis in adult DT. In hypoxia, the expression of PP2C on protein level increased (Neumann et al. 2021). In ischemic human heart failure, the expression of PP2C was enhanced (Neumann et al. 2021). Likewise, after 5 days of coronary occlusion in dogs, the expression of PP2C was increased the ischemic cardiac area (Neumann et al. 2021).

In the clinical situation, we usually assess the function of the left ventricle as it is crucial for the maintenance of the circulation. In order to assess the systolic function and to diagnose diastolic heart failure, EF is classically assessed in living mice (or patients). Therefore, we started to measure EF in the present animal models. Moreover, we usually injected intraperitoneally isoprenaline, a measure of the exercise capacity of the heart under defined stressful conditions. It is apparent that the systolic function of DT hearts is impaired under basal conditions and that they do not adequately respond to stress due to an acute injection to isoprenaline. This pattern is substantiated by quantitative measurements of several echocardiographic parameters. Moreover, the data are consistent with our previous work which indicated that PP5-TG exhibit a milder reduction of systolic and diastolic cardiac performance than PP2C. Here, the difference to DT is clear: they nearly always perform worse (in systolic and diastolic function) than PP5-TG and even than PP2C-TG and clearly are always in diminished performance compared to WT.

As expected, the expressional levels of PP2C and PP5 were higher in PP2C-TG mice or PP5 -TG than in WT on protein level and mRNA level as we published for these mice before (Bollmann et al. 2021; Dörner et al. 2021). A novel finding was a combined overexpression of PP2C and PP5-TG in DT. Interestingly, the overexpression of PP2C and PP5 was to a different extent on mRNA or protein level. This might indicate a subtle downregulation.

Cardiac hypertrophy is well known to be accompanied with elevated biochemical parameters some of which are used clinically to assess the severity of heart failure in patients. Consistent with this previous knowledge, it is plausible that the mRNA for ANP is elevated of DT hearts.

Our data are consistent with a role of inflammation with the initiation of heart failure in DT: parameters higher in DT than in the other phenotype. Hence, one might speculate that the inflammation drives heart failure in DT. In order to get closer to the mechanism of action of the overexpressed PP in DT, we looked for known substrates of PP2C and/or PP5 in vitro or in vivo. Of special importance is the reduced phosphorylation state and thence activity of PLB (Fig. 9, Neumann et al. 1999). Reduced phosphorylation of PLB can easily explain why relaxation is hindered in DT: dephosphorylated PLB inhibits the activity of SERCA (Schwinger et al. 1999); thus, more time is required to pump Ca^2+^ back into the SR and thence, Ca^2+^ remains elevated longer near the myofilaments and thus contraction takes longer and relaxation is impaired. The mechanism proposed is consistent with elevated PP2C activity in PP2C-TG and DT.

The PP5 contains only one protein sequence that contains regulatory domains and the catalytic domain (Kang et al. 2001). Interestingly, PP5 in this sequence contains an autoinhibitory domain near the N-terminus. There is a heat shock protein binding domain that is autoinhibitory and inhibits the C-terminal phosphatase domain when it moves in that direction (Liu et al. 2015; Neumann et al. 2021). This leads to an inactive form of PP5. In ischemia, in the heart, Mn^2+^ and Mg^2+^ can increase in the heart. These cations can then activate phosphatase activity of PP5. Moreover, ischemia can activate cardiac proteases. These proteases can stimulate the phosphatase activity of PP5. Lipids like arachidonic acid, heat shock proteins, S100, and Galpha_12_ can activate the phosphatase activity of PP5 (Silverstein et al. 1997). The catalytic domain of PP5 is very similar to the catalytic subunit of PP1 and to the catalytic subunit of PP2A. Organic molecules like cantharidin or okadaic acid inhibit the phosphatase activity of PP1, PP2A, and PP5 (Chen et al. 1994; Chinkers 2001). PP5 was initially found in the nucleus and later only in the cytosol of the cells (Borthwick et al. 2001, Kang et al. 2001). PP5 can bind to glucocorticoid receptors, and there, PP5 can dephosphorylate glucocorticoid receptors and alter their function (Wang et al. 2007). The activity of PP5 can even be enhanced by receptors. For instance, β_2_-adrenceptors via increasing lipid concentrations can activate PP5 in the heart (Pavione et al. 2003). Cardiac relevant substrates of PP5 include the ryanodine receptor, the L type Ca channels, TnI, phospholamban, and phosphodiesterases (Hinds et al. 2008, Neumann et al. 2021). PP5 has a tendency to be reduced in the human atrium with aging (Gergs et al. 2019b). PP5 expression was increased in the failing human heart (Krysiak et al. 2018). We have overexpressed PP5 in cardiomyocytes of transgenic mice (Gergs et al. 2012). These mice exhibited reduced cardiac contractility (Gergs et al. 2012). We have crossbred PP5 with mice which overexpress the catalytic subunit of PP2A and noted pronounce hypertrophy, increased mortality, and reduced contractility (Gergs et al. 2019a).

Similar to but different from PP5 are some properties of the PP2C. Like PP5 also PP2C and unlike PP2A, PP2C has no binding proteins that we currently know of (Neumann et al. 2021). PP2C is comprised like PP5 of a single protein chain with catalytic and regulatory domains. Like PP5 also PP2C is activated in vitro and conceivably in the beating human heart by Mn^2+^ and Mg^2+^ but inhibited by Ca^2+^ (Hu et al. 2020). PP2C is expressed in all cell types in humans and thence also in human cardiomyocytes (Seroussi et al. 2001). Like PP5 also PP2C is autoinhibitory by folding and hiding its catalytic domain. This folding can be reversed by lipids. Thereby, PP2C is activated as a phosphatase. In contrast to PP5 (or PP1 and PP2A), PP2C is not inhibited by okadaic acid (Peshenko et al. 2004). Substrates for PP2C are mainly kinases that induce proliferations. Other cardiac proteins dephosphorylated by PP2C include all proteins that are dephosphorylated by PP5 (vide supra). PP2C expression at least in fibroblasts declines upon ageing (Park et al. 2014). We generated mice that overexpress PP2C. They exhibited hypertrophy and this cardiac hypertrophy was further enhanced by crossbreeding them with mice with cardiospecific overexpression of the catalytic subunit of PP2A (Bollmann et al. 2016, 2019, 2021). PP2C may be protective in the heart against hypoxia (Bollmann et al. 2021). PP2C seemed to protect against isoprenaline-induced cardiac dysfunction (Bollmann et al. 2021).

One might hypothesize that in patients where there is overexpression of PP5 an additional overexpression of PP2C might further augment hypertrophy and increase mortality. Moreover, we suggest that drugs that reduce such elevated PP activities might be a further step into personalized drug treatment. But this remains to be elucidated.

## Limitations of the study

This study was not designed to detect sex differences between genotypes of the studied mice. For that purpose, the number of animals studied was too low and also too unevenly distributed for meaningful statistical testing. As we allocated sex at random to various study groups, this led by chance to an unequal sex distribution between experimental protocols and experimental groups. This random design also led to notable differences in the number of hearts between genotypes in the Langendorff heart perfusion studies. Moreover, in some cases, we present mRNA data whereas corresponding protein expression data are lacking. Nevertheless, in our eyes, this study adds to our knowledge on the functional role of phosphatases in the heart. This may lead to subsequent clinical studies in heart failure patients where the individual correlation of cardiac hypertrophy and phosphatase subtype expression might be interrogated.

In summary, we have shown a functionally detrimental interaction of PP2C and PP5 in the mammalian heart.

## Data Availability

The data of this study are available from the corresponding author upon reasonable request.
